# TINU-associated Fanconi syndrome: a case report and review of literature

**DOI:** 10.1186/s12882-018-1077-0

**Published:** 2018-10-19

**Authors:** Bernard Vô, Jean Cyr Yombi, Selda Aydin, Nathalie Demoulin, Halil Yildiz

**Affiliations:** 1 Department of internal medicine and infectious diseases, Cliniques Universitaires Saint-Luc, Université Catholique de Louvain, 10 Av. Hippocrate, 1200 Brussels, Belgium; 2 Department of pathology, Cliniques Universitaires Saint-Luc, Université Catholique de Louvain, 10 Av. Hippocrate, 1200 Brussels, Belgium; 3 Division of Nephrology, Cliniques Universitaires Saint-Luc, Université Catholique de Louvain, 10 Av. Hippocrate, 1200 Brussels, Belgium; 4 Institut de Recherche Expérimentale et Clinique, Cliniques Universitaires Saint-Luc, Université Catholique de Louvain, 10 Av. Hippocrate, 1200 Brussels, Belgium

**Keywords:** Tubulointerstitial nephritis and uveitis, Nephritis, interstitial, Uveitis, Fanconi syndrome

## Abstract

**Background:**

Tubulo-interstitial Nephritis and Uveitis (TINU) syndrome is a rare oculo-renal inflammatory disease. Renal tubular defects are usually found, but full proximal tubular abnormalities have rarely been described.

**Case presentation:**

We report the case of a 55-year old woman, native from Morocco, presenting with bilateral, non-granulomatous, anterior uveitis, mild renal insufficiency, leucocyturia and glycosuria. Further work-up showed hypophosphatemia and hyperphosphaturia, hypouricemia and hyperuricosuria, and hyper aminoaciduria, consistent with Fanconi syndrome. A kidney biopsy was obtained and showed diffuse interstitial infiltrates with tubular necrosis. The patient improved after the initiation of a corticosteroid therapy, with tapering dose.

**Conclusions:**

We reviewed the literature and found nine similar cases. This association mostly occurs in adult woman, without current evidence for an ethnic predilection, unlike previously reported. The renal prognosis seems favorable after corticosteroid therapy, even in case of severe renal injury. Nonetheless mild tubular defects may persist after treatment or spontaneous remission.

## Background

Tubulo-interstitial Nephritis with Uveitis (TINU) syndrome was first described in 1975 by Dobrin et al. It is defined by the occurrence of tubulointerstitial nephritis and uveitis, in the absence of another systemic condition, in accordance with Mandeville’s criteria [1]. Inflammatory and infectious diseases such as sarcoidosis, Sjögren’s syndrome, toxoplasmosis, tuberculosis, syphilis, antineutrophil cytoplasmic antibody (ANCA) associated-vasculitis and Beçhet disease should be ruled out [1, 2]. It remains a rare auto-immune disease, accounting for 0.1 to 2% of uveitis in specialized centers [2, 3] and for 4.7% of patients with acute interstitial nephritis (AIN) [4]. Its true prevalence is unknown, as many TINU may be misdiagnosed. Eye and renal manifestations may not coexist at the same moment [2], therefore emphasizing the need of close follow-up for so-called drug-induced or idiopathic AIN and uveitis, as mentioned by Su et al. [5]. The disease occurs mainly in children and adolescent [3, 4], with a median age at onset of 15 years (range 9–74 years) [1], and a female preponderance (2.5/1 to 5/1) [2, 3, 6].

TINU seems to result from a combination of host predisposition and environmental triggers [2]. To date, no compelling genetic susceptibility has been found [3], even though there are evidences concerning genetic associations, as reported by familial clusters [7] and some HLA-haplotypes [2, 3]. A close relation of TINU with antibiotics and non-steroidal anti-inflammatory (NSAID) has been mentioned. Cellular and humoral auto-immunity is suspected to play a major role in the pathogenesis of the disease [2, 8, 9]. The modified C-reactive protein (mCRP), a common uveal and renal antigen, has been recently suspected to be the target of both immune mechanisms [8].

The typical ocular manifestation is an anterior bilateral uveitis of sudden-onset, occurring 2 months before the renal presentation and up to 12 months after [1]. Nevertheless, almost every component of the uveal tract may be involved [2]. Tubulo-interstitial nephritis (TIN) usually manifests with acutely elevated creatinine level and may present with sterile pyuria or active sediment (red blood cells), tubular proteinuria and low-grade albuminuria or glycosuria. There may be renal tubular defects on urinalysis, but complete proximal tubular anomalies have rarely been described. We report a case of TINU syndrome, with features of Fanconi syndrome and performed a review of the literature about this unusual association.

## Case presentation

A 55-year-old previously healthy woman, without family history, native from Morocco, living in Belgium for almost two decades, presented at the ophthalmologist consultation with sudden onset bilateral painful red eyes and photophobia. No other current or previous complain was observed and her general state was preserved. She had no treatment, especially no NSAID, nor antibiotic. On eye examination, she was diagnosed with bilateral anterior uveitis, without granuloma. Her physical exam was otherwise normal. She benefited from an intravitreal injection of celestone and was put under degressive (1-month) topical corticosteroid therapy (prednisolone), in association with a cycloplegic agent. Laboratory tests revealed hemoglobin level 11 g/dl (NV 12.2–15), mean corpuscular volume 84.2 fl., creatinine level 1.37 mg/dl (NV 0.6–1.3), GFR (CKD-EPI) 43 ml/min/1.73m^2^ and serum potassium 3.47 mmol/l (NV 3.5–5 mmol/L). White blood cells (WBC) count showed leukocytosis to 10,830/mm^3^ (NV 4000–10,000) with neutrophils 7450/mm^3^ (NV 1600–7000) and eosinophils 630/mm^3^ (NV 30–600). Serum lysozyme, angiotensin convertase and HLA-B27 haplotype were negative, as were infectious serologies for syphilis, toxoplasmosis, HBV, HCV and HIV, and tuberculin skin test. No auto-immune marker (ANCA, antinuclear antibody and rheumatoid factor) was found. A urinary dipstick showed protein (2+), glucose (2+) and leucocytes (3+, 169/field), while the urine culture remained sterile. Laboratory tests, performed two years and one month before the initial ocular presentation, respectively demonstrated creatinine to 0.61 mg/dl and 1.08 mg/dl. Moreover, a fasting glycaemia of 91 mg/dl and a mild elevation of C - reactive protein (CRP) were found on the later.

**Fig. 1:**
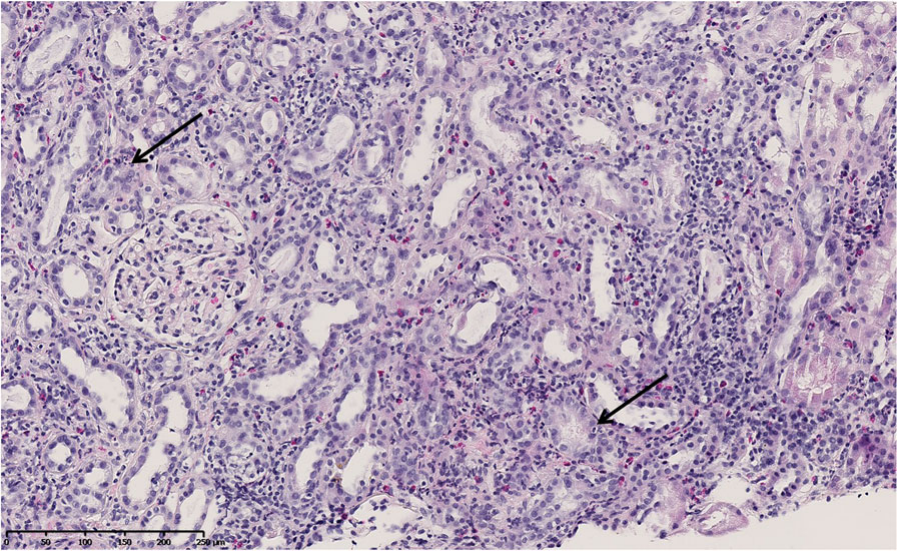
Light microscopy showing diffuse interstitial inflammation composed of mononuclear cells, polynuclear neutrophils and polynuclear eosinophils, with tubular injuries (tubulitis and acute tubular necrosis) (Arrows). No granuloma was found. The glomerulus appears damage-free (hematoxylin and eosin, original magnification X150)

The patient was addressed to our internal medicine consultation for renal evaluation. Renal involvement was confirmed (Creat. 1.14 mg/dl, GFR 54 ml/min/1.73m^2^), with hypouricemia (1.9 mg/dl; NV 2.4–5.7), hypophosphatemia (0.57 mmol/l; NV 0.84–1.45), normalized serum potassium and WBC count, normal albumin and CRP level of 16 mg/l (NV < 5). Serum protein electrophoresis and C3, C4 levels were unremarkable. Glycosuria (3.40 g/l; NV 0–0.05), proteinuria (Protein-Creatinine Ratio (PCR) 1.36 g/g) with mild albuminuria (ACR 380 mg/g) and elevated urine Beta-2 microglobulin (> 4 mg/l) were attested on the urine spot. There was no urinary free light chain, but hyperaminoaciduria. Excretional fraction for potassium was 23% (NV 10–20), uric acid 22% (NV < 10%), and phosphor reabsorption rate was 78% (NV: 85–95). Blood and urine tests showed renal proximal tubulopathy consistent with renal Fanconi syndrome. The chest radiography and renal doppler ultrasound were normal. A renal biopsy (Fig. 1.) was performed and revealed a total of 9 glomeruli (all normal, except for one sclerotic glomerulus) and severe diffuse interstitial inflammatory infiltrates with mononuclear cells, polynuclear neutrophils and eosinophils. No granuloma was identified. Foci of acute tubular necrosis and tubulitis were found. Immunofluorescence staining was negative for immunoglobulin, complement (C3d and C1q) or light chain. As the 18-FDG-PET-CT showed no focal or systemic increased uptake, we concluded to a Tubulo-Interstitial Nephritis with Uveitis (TINU) syndrome.

Soon after the diagnosis was obtained, the patient relapsed a bilateral anterior uveitis and topical steroids were restarted. Considering the severe renal involvement found on biopsy, an oral degressive-corticosteroid therapy (initial dose, methylprednisolone 48 mg/d; tapered over 8 months) was initiated. This resulted in a favorable renal outcome at six weeks (creat. 0.89 mg/dl, GFR 73 ml/min/1.73m^2^, no glucosuria, serum uric acid 2.4 mg/dl), but with persistent leucocyturia.

## Discussion and conclusions

TINU has been associated with glycosuria, proteinuria and others proximal tubular dysfunction [1, 2], but has rarely been described with a full proximal defect. Fanconi syndrome can be either inherited (e.g. cystinosis, tyrosinemia, Wilson’s disease, Lowe’s syndrome, Dent disease, …) [10] or acquired, resulting from diseases such as light chains secreting monoclonal gammapathies, amyloidosis, Sjögren syndrome, drugs (aminoglycoside, ifosfamid, cisplatin, tenofovir, …), or toxic (arsenic, cadmium, lead, mercury) [11]. We assume that those situations should be first ruled out in case of biological criteria for Fanconi syndrome.

We searched the MEDLINE database, using PubMed, for reports concerning TINU and Fanconi syndrome, between 1975 and January 2018, mentioning Mesh terms “Fanconi syndrome”, “Nephritis” and “Uveitis”. Additional publications were identified by screening the references. Koike et al. previously performed a literature review. One of the patients was reported in the Japanese literature [12], so that we used the information listed in Koike’s review. We found four supplementary cases, resulting in a description of 10 cases [4, 6, 9, 12–16], including ours. Among these, two were included from the Legendre et al.’s cohort, but with few data available. Cases attributed to other systemic diseases were excluded in accordance to Mandeville’s criteria. The clinical, biological and demographic findings are summarized in Table 1.

**Table 1:** Clinical, biological and demographic characteristics

Autor (year)	Age/sex	Origin	Initial presentation	Uveitis type	Serum creatinine (mg/dl)	Serum potassium (mmol/l)	Serum phosphate (mmol/l)	Serum uric acid (mg/dl)	Serum bicarbonate (mEq/l)	Other manifestation	Other renal manifestations	Steroid therapy	Predisposing factor (?)	Renal outcome	Uveitis relapse
Takeda (1988) [12]	45/F	Japan	NA	Bilateral iritis	1,8	4	0,94	1,8	17	NA	Metabolic acidosis, glucosuria, AA	topical	Unknown	NA	NA
Lessard (1989) [13]	48/F	White (USA)	Uveitis	Left anterior uveitis	2,5	2,9	0,55	NA	20	anorexia, malaise, fatigue, nausea, headache, chills	u pH 6,5, glucosuria, proteinuria, AA, urinary eosinophilia	topical steroids, oral prednisone (60 mg/d, 4w; gradual tapering for 16w)	Oral penicillin for toothache? High ASLO titer	Favorable, persistent hypophosphatemia	NA
Igarashi (1992) [14]	11/M	Japan	TIN	Bilateral anterior uveitis	1,2	3,5	1,16	1,3	16,3	malaise, asthenia, weight loss	u pH 7,36, metabolic acidosis, distal tubular dysfunction, glucosuria, proteinuria, AA	topical steroids	Unknown	Favorable, normal creat. (13 months), normalization of tubular function	NA
Wakaki (2001) [9]	13/F	Japan	Uveitis	Bilateral anterior uveitis	1,1	3,3	0,83	1,8	25	fatigue, abdominal pain, weight loss	u pH 7.5, distal tubular dysfunction, glucosuria, proteinuria, leucocyturia, urinary eosinophils	topical steroids	Auto-antibody to renal tubular cells	Favorable, creat. 0,6 (6 months), normalization of tubular function	NA
Koike (2006) [4]	32/F	Japan	TIN	Bilateral iritis	3,39	3,2	0,9	2,8	19,4	fatigue, anorexia, weight loss, fever	u pH 6.5, metabolic acidosis, microscopic hematuria, glucosuria, proteinuria, AA, leucocyturia	topical steroids, oral prednisolone (0.8 mg/kg/d)	levofloxacin for fever, possible streptococcal infection (ASLO +)	Favorable, creat. 0,59 (36 weeks), persistent mild hypokalemia	Yes (5 months)
Yao (2009) [15]	57/F	Taiwan	Uveitis	Bilateral anterior uveitis	1,95	3	0,8	2,2	15,7	NA	Metabolic acidosis, glucosuria, proteinuria	Oral methylpred. (16 mg/d)	NSAID for herpes zoster infection	Favorable, creat. 1,39 (20 days), persistent glucosuria	NA
Llorente (2012) [16]	9/M	Spain	TIN	Anterior uveitis	1,2	4,3	1,4	2,1	17,5	fatigue, anorexia, weight loss, fever, enuresis, hypertension	Metabolic acidosis, glucosuria, proteinuria, leucocyturia	topical steroids	Unknown, high ASLO titer	Favorable, creat. 0,7 (30 weeks), persistent metabolic acidosis	Yes
Legendre (2016) [6]	23/F	NA	NA	NA	1,71	NA	NA	NA	NA	NA	NA	1 mg/kg/d	NA	Favorable, creat 1.15 (30 months)	NA
Legendre (2016) [6]	46/F	NA	NA	NA	1,78	NA	NA	NA	NA	NA	NA	0.5 mg/kg/d	NA	Favorable, creat 0.6 (30 months)	NA
Present case	55/F	Morocco	TIN	Bilateral anterior uveitis	1,14	3,47	0,57	1,9	25	None	u pH 7, glucosuria, AA, proteinuria, leucocyturia	topical steroids, methylpred. (48 mg/d)	Unknown	Favorable, creat. 0,89 (6 weeks), persistent leucocyturia	Yes (1 month; before oral steroids treatment)

Abbreviations: *M* male, *F* female, *TIN* tubulo-interstitial nephritis, *NA* not available, *u pH* urinary pH, *AA* aminoaciduria

Seven cases occurred in adult women, all three others in children, including two males. The median age is between 32 and 45-year old (mean age 34). Four patients were from Japan and one from Taiwan, but other origins were also described. The initial presentation leading to diagnosis could be either TIN or uveitis. Renal manifestations included moderate to severe renal insufficiency, with arguments for proximal tubular defects (glycosuria, aminoaciduria, tubular proteinuria and metabolic acidosis). Hypokalemia was mostly mild and serum phosphate was lowered in half of cases. Serum uric acid was low in all but one description available and serum bicarbonate was diminished in six patients. Leucocyturia was a common finding on urinalysis. Frequent systemic manifestations were present and the most cited complains were fatigue and weight loss. Two patients benefited from previous antibiotherapy (suspected oral infection and fever) and one from NSAID. Anti-streptolysin O (ASLO) titer was elevated in three cases without manifest streptococcal infection and one presented with recent herpes zoster infection. Wakaki et al. observed positive auto-antibodies to renal tubular cells. Most patients benefited from topical steroids to relieve the eye symptoms. Oral steroids were introduced in six patients. Reasons for steroid therapy were mentioned in three cases, two for severe renal damage and one for uveitis. The renal function improved in all cases, but some tubular defects could persist at a milder degree.

Koike et al., predominantly reported the association of TINU and Fanconi syndrome in adult Japanese women. They advanced the hypothesis of thorough initial evaluation due to high medical expenses coverage, allowing larger diagnosis of the disease in the Japanese population compared to others. They further noted that HLA-A2 and HLA-A24 haplotypes, mostly associated with TINU, have a high prevalence in Japan. Nonetheless, this association has not been confirmed [3]**.** Since then, additional cases have been described in other populations, including Taiwanese, Spanish and Moroccan, favoring the absence of racial predilection, in concordance with observations made in the larger population of TINU.

No causative agent appears to be associated with TINU and Fanconi syndrome. The classical environmental factors, NSAID and antibiotic, observed in TINU, were also found in the population of our review. Evidences of humoral and T-cell mediated immunity activation were reported, as previously mentioned. However, whether auto-antibodies to tubular cell, as found by Wakaki et al., participate to the pathogenesis of Fanconi syndrome and TINU or are secondary to exposure of tubular antigens, has not been established.

The use of systemic corticoid for AIN remains debated. Both topical and oral steroids are employed in the TINU population, to relieve eye symptoms or to treat AIN, even though spontaneous remissions have been noted. The clinical course of eye and kidney manifestations seems to be independent. It is accepted to use oral prednisolone at an initial dose of 1–1.5 mg/kg/d for those with progressive renal impairment, or severe, posterior or relapsing uveitis, although this is not resulting from an evidence based approach [2, 17]. The schedule for degressive corticosteroid therapy depends on the clinician decision and relies on the patient’s response. Other immunosuppressive agent may be used [2]. Patients suffering from TINU used to have favorable prognosis [1]. Recently, studies in adult cohorts showed contradictory results with persistent renal dysfunction at middle to long-term follow-up [6, 18]. In our review, patients with severe renal involvement benefited from a systemic therapy. This resulted in significant improvement in renal function and in proximal tubular abnormalities, but with short term follow-up. Notwithstanding, mild persistent tubular dysfunction remained in most of the reports. In our case, we took the decision to treat the patient due to the severe interstitial infiltration and tubular necrosis found on renal biopsy and after a first relapse of uveitis. This resulted in a favorable renal outcome, with persistent leucocyturia at short term follow-up.

TINU and Fanconi syndrome constitute a rare association. Observations made in this review should be used carefully, as possible publication and diagnosis bias may co-exist, and median follow-up is short, therefore preventing definite conclusions. Nonetheless, some points may be emphasized. The association mostly arises in adult women, differing from the demographic characteristics from the overall TINU population. It occurs in more diverse populations than initially reported, without current evidence for any ethnic predilection. The renal disease seems mostly to be self-limited, and the prognosis remains good in case of severe renal injury, which encourages a systemic steroids administration, even if mild tubular defects may persist. The timing and dose of oral steroids remain to be clearly defined, as it is for duration and schedule for tapering of steroid dosage.

## Abbreviations

AIN: Acute interstitial nephritis
ANCA: Antineutrophil cytoplasmic antibody
NSAID: Non-steroidal anti-inflammatory drug
TIN: Tubulo-interstitial nephritis
TINU: Tubulo-interstitial nephritis and uveitis
WBC: white blood cell
